# Impact of *APOE* ε4 genotype on initial cognitive symptoms differs for Alzheimer’s and Lewy body neuropathology

**DOI:** 10.1186/s13195-021-00771-1

**Published:** 2021-01-23

**Authors:** Jagan A. Pillai, James Bena, Aaron Bonner-Jackson, James B. Leverenz

**Affiliations:** 1 Department of Neurology, Lou Ruvo Center for Brain Health, Cleveland Clinic Lerner College of Medicine, Case Western Reserve University, 9500 Euclid Ave / U10, Cleveland, OH 44195 USA; 2 Cleveland Clinic, Neurological Institute, Cleveland, OH 44195 USA; 3 Department of Neurology, Cleveland Clinic, Cleveland, OH 44195 USA; 4 Quantitative Health Sciences, Cleveland Clinic, Cleveland, OH 44195 USA

**Keywords:** *APOE ε4*, Dementia, Cross-sectional study, Initial cognitive symptom, Non-amnestic, Neuropathology, Lewy body, Alzheimer’s, Visuospatial, Language, Executive

## Abstract

**Background:**

*APOE ε4* carrier status is known to increase odds of amnestic presentations with Alzheimer’s pathology. It is unknown how *APOE ε4* carrier status impacts odds of specific initial cognitive symptoms in the presence of Lewy body pathology. Here we evaluate the impact of *APOE ε4* genotype on initial cognitive symptoms among those with Alzheimer’s disease pathology (ADP) and Lewy-related pathology (LRP).

**Methods:**

A retrospective cohort study of 2288 participants with neuropathology confirmed ADP or LRP in the National Alzheimer’s Coordinating Center database, who had initial cognitive symptoms documented and had a Clinical Dementia Rating-Global (CDR-G) score ≤ 1 (cognitively normal, MCI, or early dementia). Unadjusted and adjusted logistic regression models taking into account age at evaluation, sex, and education examined the relationship between *APOE ε4* genotype and initial symptoms (memory, executive, language visuospatial) among ADP with LRP and ADP-LRP groups.

**Results:**

One thousand three hundred three participants met criteria for ADP alone, 90 for LRP alone, and 895 for co-existing ADP and LRP (ADP-LRP). Younger age increased odds of non-amnestic symptoms across all three groups. In the adjusted model among ADP, *APOE ε4* carriers had higher odds of amnestic initial symptoms 1.5 [95% CI, 1.7–2.14, *p* = 0.003] and lower odds of initial language symptoms 0.67 [95% CI, 0.47–0.96, *p* = 0.03] than non-carriers. The odds for these two symptoms were not different between ADP and mixed ADP-LRP groups. Female sex and higher education increased odds of initial language symptoms in the ADP group in the adjusted model. In the unadjusted model, *APOE ε4* carriers with LRP had a higher odds of visuospatial initial symptoms 21.96 [95% CI, 4.02–110.62, *p* < 0.0001], while no difference was noted for initial executive/attention symptoms. Among LRP, the odds of *APOE* ε4 on amnestic symptom was not significant; however, the interaction effect evaluating the difference in odds ratios of amnestic symptom between ADP and LRP groups also did not reach statistical significance.

**Conclusions:**

The odds of specific initial cognitive symptoms differed between ADP and LRP among *APOE ε4* carriers compared to non-carriers. The odds of initial amnestic symptom was higher among ADP *APOE ε4* carriers and the odds of visuospatial initial symptom was higher with LRP *APOE ε4* carriers. This supports the hypothesis that *APOE ε4* differentially impacts initial cognitive symptoms together with underlying neuropathology.

**Supplementary Information:**

The online version contains supplementary material available at 10.1186/s13195-021-00771-1.

## Introduction

Alzheimer’s disease (AD) in its typical clinical presentation is well known to present with early episodic memory deficits followed by progressive impairments in other cognitive domains including visuospatial, language, and executive function as the disease progresses. The underlying pathological change noted is the increasing burden of neuritic amyloid-β plaques and neurofibrillary tangles. There has been a recognition of significant heterogeneity among early clinical symptoms different from the common amnestic presentation, with atypical non-amnestic AD phenotypes formalized in the International Working Group (IWG)-2 clinical diagnosis criteria [1]. Biomarker differences including CSF total-tau [2] and MRI imaging [3] between those with early amnestic and non-amnestic symptoms (predominant initial symptoms of language, executive, or visuospatial dysfunction) along with patterns of neurofibrillary tangle accumulation [4] suggest that there could be underlying biological characteristics that influence early presentations of the initial predominant clinical symptom.

Age and genetic status appear to play a role in the early clinical phenotype of AD. Early non-amnestic presentations of AD have been reported more often in younger-onset AD [5, 6]. The *Apolipoprotein ε4* (*APOE ε4*) allele, most frequently associated with an increased risk of late-onset AD, has been related to amnestic presentations [7–10]. Increased prevalence of *APOE ε4* allele among amnestic forms of AD has also raised the hypotheses that *APOE ε4* is an anatomically selective risk factor that increases vulnerability to AD pathology (ADP) in memory-related medial temporal regions and that it possibly modulates the clinical phenotype of AD through the influence of specific large scale brain networks [10, 11]. It has also been reported that *APOE ε4* carrier proportion was not elevated among aphasic variants of AD [10]. These findings in AD raises further questions: one, if *APOE ε4* allele differently impacts odds of other non-amnestic clinical symptoms of AD (executive, visuospatial), and two, if *APOE* ε4 carriers with a different underlying neuropathology from Alzheimer’s would also share a similar susceptibility to amnestic symptoms.

Dementia with Lewy bodies (DLB) is among the most common forms of dementia [12] and is characterized by Lewy-related pathology (LRP), including neuronal inclusions which are α-synuclein immunopositive (Lewy bodies) and processes (Lewy neurites). Many subjects with LRP also show coexistent ADP (ADP-LRP). *APOE ε4* allele is a strong risk factor across the Lewy body disease spectrum and increases the likelihood of presenting with dementia in the context of even a pure synucleinopathy without co-existent ADP [13]. Attention, visuospatial, and visuoconstructive deficits are often predominant cognitive domain differences in dementia with Lewy bodies compared to AD but they often also have variable memory deficits [14–16]. A progressive staging system of LRP relating to neuropathology and cognition in Parkinson’s disease has been proposed and DLB patients are thought to display this same sequence of cortical involvement [17, 18]. In this context, it is unknown how *APOE* ε4 carrier status would impact odds of amnestic or other non-amnestic cognitive domains including visuospatial deficits among those with LRP and with the mixed pathology of ADP-LRP.

We therefore undertook the current study in the National Alzheimer’s Coordinating Center (NACC) dataset that includes a highly ascertained cohort across multiple sites across the USA with neuropathology information available for a large proportion of participants. This makes it a unique resource to investigate clinical factors that interact with *APOE ε4* across various neuropathologies. Given the absence of detailed information to characterize atypical variants within the IWG-2 criteria in NACC, we determined to look for initial cognitive symptoms as a potential window into the primary cognitive domain likely to be affected. We had two hypotheses. Hypothesis one: *APOE ε4* carrier group would have higher odds of initial amnestic symptoms (compared to *ε4* non-carriers) regardless of the underlying neuropathology ADP alone, LRP alone, or ADP-LRP. Hypothesis two: *APOE ε4* carrier status would not increase odds of any of the non-amnestic initial symptoms (visuospatial, language, executive/attention) in the same three neuropathology groups compared to *APOE ε4* non-carriers. Baseline neuropsychology profile of participants was characterized as a secondary validation.

## Materials and methods

### Participants and study design

A retrospective cross-sectional study using the NACC dataset was conducted. The dataset used for this analysis includes participant information collected from 37 past and present Alzheimer’s Disease Centers (ADC) funded by the National Institute on Aging. Data from the Uniform Data Set (UDS) maintained by NACC between September 2005 and September 2019 was used for the present analysis. This includes participants with cognitive status ranging from normal cognition to mild cognitive impairment (MCI) and demented. All contributing ADCs are required to obtain informed consent from their participants and maintain their own separate IRB review and approval from their institution prior to submitting data to NACC. Details on data collection and data curating are well documented [19]. In brief, NACC data are collected by trained clinicians and clinic personnel from participants and their co-participants (usually a close friend or family member). The UDS is collected using a standardized evaluation of participants. All of the ADC personnel use the same standard forms and coding guidebooks that provide guidance on filling out the forms. The forms are developed by representatives from the ADCs themselves, so they are involved in the process of creating the standard forms. The UDS is longitudinal, and its protocol requires approximately annual follow-up for as long as the participant is able to be involved. Late-stage participants forced to drop out due to health may continue to be followed strictly for autopsy purposes. Determinations of cognitive status in NACC are based on a clinical consensus after a review of all available information at each center. In addition, there is evidence supporting good agreement on measures from the NACC Neuropathology form across centers used in this study [20]. NACC subjects are not a statistically based sample of the US population—with or without dementia. Rather, they are best regarded as a referral-based or volunteer case series.

### Participant assessment/inclusion criteria

All participants included in the analysis had neuropathology of AD (Tau neurofibrillary tangle pathology Braak stages III–VI and moderate or frequent neuritic plaques) or LRP (brainstem-predominant, limbic or amygdala-predominant, neocortical, or had Lewy bodies present but region unspecified). The ADP group had underlying AD pathology without concomitant LRP and the LRP group had underlying LRP without concomitant ADP. When both pathologies were documented concomitantly, it was noted as belonging to the ADP-LRP group. In addition, as mixed vascular pathology is expected in many older age subjects and the degree of vascular pathology could change over time from initial visit to autopsy, all participants were characterized for the likelihood of co-existing vascular pathology by the Hachinski ischemic scale when available at the time of initial evaluation for each group [21].

Further, all participants completed CDR® Dementia Staging Instrument score and had a Clinical Dementia Rating-Global (CDR-G) scale ≤ 1 at the initial clinical visit. The CDR-G scale assesses the participant’s current cognitive and functional status. The CDR-G ratings are calculated using a complex algorithm and range from 0 (no dementia) to 3 (severe dementia) [22]. In this regard, a CDR-G = 1 would correspond to the threshold of early dementia. Rationale for limiting analysis to those with CDR-G ≤ 1 was to help increase the reliability of data regarding initial cognitive symptoms closest to their onset when the dementia symptoms are early. Details on the number of cognitively normal, MCI, and early dementia subjects at the initial visit are provided in the supplementary material. All participants included in this analysis also had known *APOE* ε4 status and the main analysis was conducted with ε4 present (i.e., carrying one or both ε4 alleles) versus absent (carrying no ε4 alleles).

**Fig. 1:**
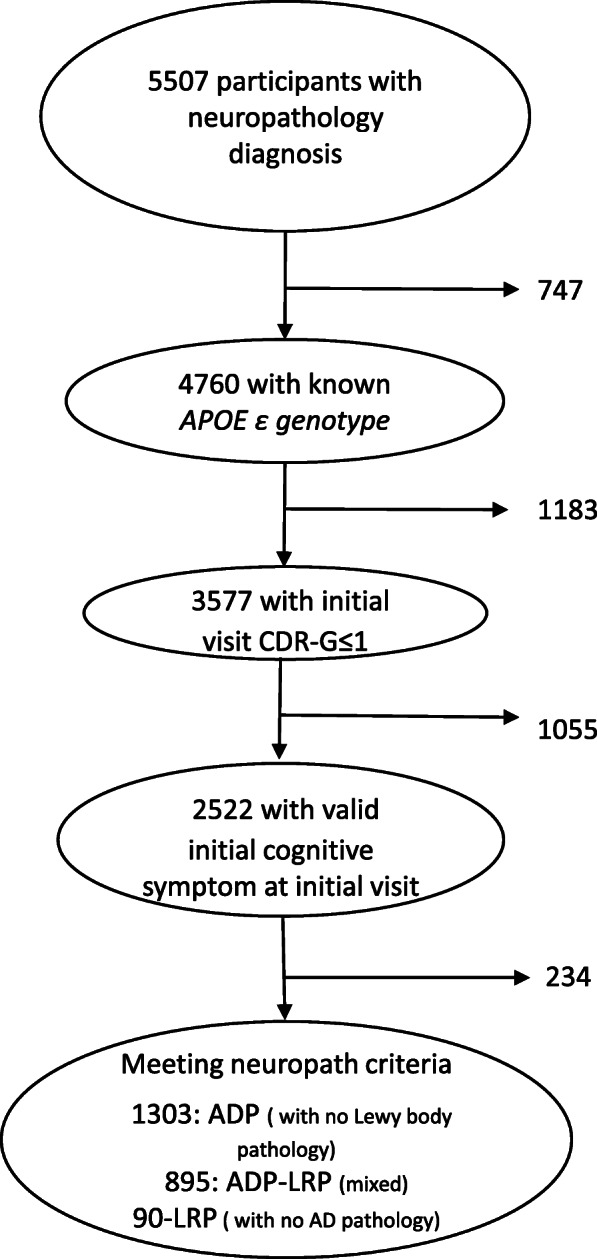
Participant selection flow chart

Figure 1 provides the subject selection flow chart.

### Initial cognitive symptoms and neuropsychological tests

“NACCCOGF” is a NACC-derived variable that indicates the predominant symptom documented by the clinician that was first recognized as a decline in the subject’s cognition. All clinicians use the same standard forms and coding guidebooks, but there is no structured interview formalized for determining the answer to this variable. Per the NACC guidelines, the clinician’s conclusions for the “NACCCOGF” variable are expected to be based on information obtained through subject, co-participant, medical records, and/or observation. Furthermore, results from the neuropsychological test battery (except for the Montreal Cognitive Assessment) and imaging are not be used to determine the answer for this question by the clinician. Specific clinical phenotypes of frontal, logopenic, and posterior variants of AD could not be defined as per the IWG-2 research diagnostic criteria based on the first cognitive domain of decline clinical impression alone as noted in NACC [1]. We therefore characterized the subjects as having primary amnestic complaints (if memory was the initial symptom), executive (if executive or attention/concentration were the initial symptoms), and visuospatial (if visuospatial dysfunction was the initial symptom). Of note, the participant’s neuropsychology data were not used in determining the initial symptoms of participants which were likely perceived by them before their initial visit. Our secondary analysis of the neuropsychology data was done to ensure that the significant cognitive deficits at the visit clinical visit were broadly congruent with the initial symptoms of the patients prior to their evaluation by the clinician.

A core battery of neuropsychological measures was administered to all participants at each visit [23]. Data from their first visit among those who had CDR-G ≤ 1 were analyzed. All four cognitive domains documented in the UDS were evaluated: attention, executive functioning, language, and memory. Attention was assessed using the Digit Span subtest (Digits Forward) from the Wechsler Adult Intelligence Scale (WAIS) [24] and the Trail-Making Test (TMT) Part A [25]. Executive functioning was quantified using WAIS Digit Span (Digits Backwards) [24], Trail Making Test Part B, and the Digit Symbol-Coding subtest from the WAIS [24, 25]. Digits Backward Length (i.e., number of digits correctly repeated in reverse order) was also included as a variable of interest. Language-related tests included in NACC were object naming assessed using the 30-item version of the Boston Naming Test (BNT) [26] and semantic fluency (animal/vegetable names generated in 60 s) [27]. The evaluation of memory included measures of verbal episodic memory (Wechsler Memory Scale, Logical Memory subtest) [24, 28]. The number of subjects with specific tests of visuospatial function in the NACC data (Benton Figure copy and draw) was not adequate for detailed analysis as it was only provided from 2015 (version 3 of UDS).

### Statistical analysis

Q-Q plots were generated for continuous variables to assess normality. A *t* test was applied to compare normally distributed continuous variables. Eta squared > 0.01 was considered a threshold effect size of significance for interpreting neurocognitive variable differences in this study. Mann-Whitney *U* test was applied to compare non-normally distributed continuous variables. Chi-squared test was conducted for categorical variables. Unadjusted odds ratios were calculated first using chi-squared test or Fisher’s exact test (when the cell count was 10 or lower). When the cell count allowed, adjusted logistic regression analyses were conducted with age, sex (male, female), and education (years) included as covariates given the differences between ADP and LRP groups.

Two models were run for each combination (model 1: ADP versus LRP and model 2: ADP versus ADP-LRP). Differences between the neuropathology groups by *APOE* ε4 status and *APOE* ε4 effect within each group were evaluated. The models characterized two key effects (main effect for *APOE* ε4 carrier status on an initial cognitive symptom and *APOE* ε4 X neuropathology group interaction). The *APOE* ε4 X neuropathology group interaction effect assesses the strength of whether the effect of *APOE* ε4 carrier status on initial cognitive symptom differs between two neuropathology groups (ADP versus LRP and ADP versus ADP-LRP). The other covariates included age, sex, and education years.

As planned comparisons used to evaluate the effect of *APOE* ε4 in each group were decided a priori and every possible comparison is not being evaluated, multiple comparison correction was not applied and individual *p* values for comparisons are provided in results.

To test the utility of APOE ε4 dose (0, 1, and 2) in impacting logistic regression model results against (APOE-ε4 present/absent), we compared both the models based on the Vuong test, (R package, pscl v1.5.5). All tests were two-tailed and performed at a significance level of 0.05. R version 3.5.1 (The R Foundation for Statistical Computing, Vienna, Austria) and IBM SPSS Statistics for Windows, Version 22.0. Armonk, NY: IBM Corp were used for all analyses.

## Results

The demographics of participants in the cohort are described in Table 1. The LRP group was younger with a male predominance and a lower proportion of *APOE ε4* carriers compared to ADP and ADP-LRP groups. In addition, the LRP group had proportionally more subjects with non-amnestic initial symptoms and a lower proportion of amnestic symptoms at initial presentation compared to ADP and ADP-LRP groups. On comparing the Hachinski ischemic scale scores to determine the relative differences between the initial symptom groups (e.g., amnestic versus non-amnestic) on coexisting vascular symptoms, the mean scores across all groups were < 2 and the scores were not significantly different between the initial symptom groups (Supplementary Tables 1, 2, and 3).

**Table 1:** Participant demographics

	Alzheimer’s pathology (ADP)		Lewy-related and Alzheimer’s (ADP-LRP)		Lewy-related pathology (LRP)			
	***N***	Mean (Std. Dev) or % of total	***N***	Mean (Std. Dev) or % of total	***N***	Mean (Std. Dev) Or % of total	***F***/***χ***^**2**^	Sig.
**Age at first visit, years**	1303	76.35 (10.56)	895	73.79 (10.07)	90	73.3 (9.27)	17.9	< 0.0001
**Duration from first visit to autopsy, years**	1303	4.93 (2.79)	895	5.39 (2.89)	90	4.36 (2.82)	11.29	< 0.0001
**Age at death, years**	1303	81.28 (10.40)	895	79.08 (10.17)	90	77.67 (8.50)	17.3	< 0.0001
**Education, years**	1296	15.34 (3.07)	889	15.5 (3.08)	90	15.66 (3.07)	0.95	0.39
**Sex % F**	1303	45.60%	895	37.40%	90	25%	24.47	< 0.0001
**APOE ε4% positive**	1303	52.60%	895	61.60%	90	20%	62.98	< 0.0001
**Amnestic**	1297	80.40%	890	81.10%	89	58.40%	26.5	< 0.0001
**Executive/attention**	1297	5.86%	890	6.51%	89	11.20%	4.15	0.13
**Language**	1297	11.50%	890	9.21%	89	20.20%	11	0.004
**Visualspatial**	1297	2.20%	890	3.10%	89	10.10%	18.7	0.002

### Unadjusted model results

*APOE* ε4 carrier status did not significantly impact the odds of any of the initial cognitive symptoms for ADP (significance level for amnestic symptoms was *p* = 0.054). In the ADP-LRP mixed pathology group, *APOE* ε4 increases the odds of initial amnestic symptoms by 1.56 (95% confidence interval [CI] 1.11–2.19, *p* = 0.01) and decreases odds of language initial symptoms by 0.50 (95% confidence interval [CI] 0.32–0.79, *p* = 0.003). Among LRP, the odds of *APOE* ε4 on amnestic symptoms was not significant.

*APOE* ε4 increased the odds of initial visuospatial symptoms in LRP by 21.96 (95% confidence interval [CI] 4.02–110.62, *p* < 0.0001). Confidence limits reflect wide variability for this estimate given the smaller number of LRP subjects (*n* = 90) and those with initial visuospatial symptoms (*n* = 9) among them. Table 2 summarizes the significant odds ratio for all three pathologies in the unadjusted models.

**Table 2:** Unadjusted odds ratio and 95% confidence intervals from chi-squared or Fisher’s exact tests across ADP, ADP-LRP, and LRP groups for specific initial cognitive symptoms and *APOE ε4* genotype. Logistic regression model with *APOE e4* is the exposure, initial cognitive as outcomes, and pathology as subgroups

**ADP**	**Total**	**APOE ε4** **-ve**	**APOE ε4** **+ve**	**Chi square** ***p*** **value**	
Amnestic	1043	480	563	*p* = 0.054	
	80.40%	78.20%	82.40%		
Executive/Attention	76	36	40	*p* = .996	
	5.90%	5.90%	5.90%		
Language	149	79	70	*p* = 0.14	
	11.50%	12.90%	10.20%		
Visualspatial	29	19	10	*p* = 0.059*	
	2.20%	3.10%	1.50%		
*Fischer’s exact p value presented as small cell count					
**LRP-ADP**	**Total**	**APOEε4** **-ve**	**APOEε4** **+ve**	**Chi square** ***p*** **value**	**Odds ratio**
Amnestic	722	262	460	*p* = 0.01	1.56(1.11-2.19)
	81.10%	77%	84%		
Executive/Attention	58	23	35	*p* = 0.83	
	6.50%	6.70%	6.40%		
Language	82	44	38	*p* = 0.003	0.5 (0.32-0.79)
	9.20%	12.90%	6.90%		
Visualspatial	28	12	16	*p* = 0.62	
	3.10%	3.50%	2.90%		
**LRP**	**Total**	**APOEε4** **-ve**	**APOEε4** **+ve**	**Fishers exact** ***p*** **value**	**Odds ratio**
Amnestic	52	43	9	*p* = 0.29	
	58.40%	60.60%	50%		
Executive/Attention	10	9	1	*p* = 0.68	
	11.20%	12.70%	5.60%		
Language	18	17	1	*p* = 0.1	
	20.20%	23.90%	5.60%		
Visualspatial	9	2	7	*p* < 0.0001	21.96(4.02-110.62)
	10.10%	2.80%	38.90%		

### Logistic model results

#### Model 1 (ADP versus LRP)

In the adjusted models, when evaluating among ADP and LRP participants, adjusting for age, sex, and education, *APOE* ε4 increases odds of initial amnestic symptoms in the ADP group by 1.58 times (95% CI 1.17–2.14, *p* = 0.0031) compared to *APOE* ε4 non-carriers. The odds of amnestic symptoms in LRP did not reach statistical significance among *APOE* ε4 carriers. However, the interaction effect of *APOE* ε4 X ADP and *APOE* ε4 X LRP groups (evaluating the difference in the odds ratios of amnestic symptom between the neuropathology groups) did not reach statistical significance (Tables 3 and 4). The parameter estimates of all adjusted models are provided in Supplementary Table 4.

**Table 3:** Adjusted odds ratio and 95% confidence intervals for model 1 (ADP versus LRP) and model 2(ADP versus ADP-LRP). Logistic regression model with *APOE e4* is the exposure, initial cognitive as outcomes, and pathology as subgroups and age, sex, and education as covariates. Significant results (*p* < 0.05) are in bold. Model 1: ADP versus LRP

	Amnestic	Executive/attention concentration	Language	Visualspatial
***APOEε*4 odds in ADP**	**1.58 (1.17–2.14)**	0.99 (0.61–1.60)	**0.67 (0.47–0.96)**	0.46 (0.20–0.99)
**Age at visit**	**1.1 (1.08–1.1)**	**0.93 (0.92–0.96)**	**0.93 (0.92–0.95)**	**0.92 (0.89–0.95)**
**Sex, female**	0.75 (0.56–1.01)	0.73 (0.45–1.17)	**1.71 (1.2–2.4)**	0.99 (0.47–2.03)
**Education, years**	0.96 (0.91–1.01)	1.0 (0.92–1.08)	**1.07 (1.01–1.14)**	1.0 (0.89–1.13)

Adjusted odds ratio for LRP as a reference not presented due to smaller cell counts

**Table 4:** Adjusted odds ratio and 95% confidence intervals for model 1 (ADP versus LRP) and model 2(ADP versus ADP-LRP). Logistic regression model with *APOE e4* is the exposure, initial cognitive as outcomes, and pathology as subgroups and age, sex, and education as covariates. Significant results (*p* < 0.05) are in bold. Model 2: ADP versus ADP-LRP

	Amnestic	Executive/attention concentration	Language	Visualspatial
***APOEε*4 odds in ADP**	**1.86 (1.3–2.7)**	0.88 (0.51–1.54)	**0.44 (0.27–0.70)**	0.78 (0.36–1.73)
***APOEε*4 odds in ADP-LRP**	**2.28 (1.05–4.97)**	0.78 (0.24–2.64)	**0.28 (0.10–0.77)**	1.36 (0.24–8.00)
**Age at visit**	**1.07 (1.06–1.09)**	**0.95 (0.93–0.96)**	**0.95 (0.93–0.96)**	**0.93 (0.91–0.95)**
**Sex, female**	0.44 (0.85–1.37)	**0.65 (0.44–0.95)**	1.14 (0.85–1.53)	0..94 (0.53–1.64)
**Education**	**0.94 (0.90–1.36)**	1.01 (0.95–1.07)	**1.1 (1.05–1.16)**	1.03 (0.89–1.14)

*APOE* ε4 decreased the odds of initial language symptoms in ADP by 0.67 (95% CI 0.47–0.96, *p* = 0.03) that is a 33% decrease in the odds among *APOE* ε4 carriers with ADP, compared to *APOE* ε4 non-carriers with ADP. Again, for the interaction effect of *APOE* ε4 X ADP and *APOE* ε4 X LRP groups, the difference in the odds ratios between the neuropathology groups did not reach statistical significance.

There was no significant main effect of *APOE* ε4 on executive and visuospatial symptoms in ADP. The adjusted logistic regression models were of limited utility in LRP for executive and visuospatial initial symptoms due to wide confidence limits given the smaller participant numbers and are not presented. Female sex and higher education were also significant factors for increasing odds of language as an initial symptoms in the logistic model comparing ADP and LRP (Tables 3 and 4).

#### Model 2 (ADP versus ADP-LRP)

*APOE* ε4 increases odds of initial amnestic symptoms in the mixed ADP-LRP group by 2.28 times (95% CI 1.05–4.97, *p* = 0.036) compared to non-carriers. The odds of language symptoms among *APOE ε4* was also lower in ADP-LRP mixed group 0.28 (95% CI 0.10–0.77, *p* = 0.01), a 72% decrease in odds compared to *APOE* ε4 non-carriers. There was no significant effect of *APOE* ε4 carrier status on executive and visuospatial symptoms for ADP and ADP-LRP groups. The interaction effect of *APOE* ε4 X ADP-LRP and *APOE* ε4 X ADP groups (evaluating the difference in odd ratios of amnestic symptom between these neuropathology groups) did not reach statistical significance (Tables 3 and 4).

Female sex was a significant factor for decreasing odds of executive/attention as initial symptoms in the same model 2, while higher education decreased odds of amnestic initial symptoms and increased language symptoms in the same model (Tables 3 and 4).

Younger age was a significant factor for increasing odds of non-amnestic initial symptoms with ADP, ADP-LRP, and LRP in both models 1 and 2.

### Supplementary analyses

#### Neurocognitive profile results

We next explored the neurocognitive profile among a subset of participants with concomitant neurocognitive scores at the first visit as a secondary validation of initial cognitive symptoms (often measured after the onset of initial cognitive symptom). Supplementary Tables 1, 2, and 3 show the comparative neuropsychology test performance profile of participants with the four initial cognitive symptoms evaluated in this study for ADP, ADP-LRP, and LRP groups. The number of subjects with completed neurocognitive scores was lower in the LRP group compared to AD and ADP-LRP groups, limiting their statistical significance results.

The amnestic groups had lower logical memory delayed scores with notable effect sizes compared to non-amnestic groups for ADP and ADP-LRP, while the non-amnestic group performed significantly worse on tests of attention and executive function in ADP and ADP-LRP groups.

Subjects with initial executive/attention symptoms performed lower on Digit Span tests for ADP and ADP-LRP and better on logical memory delayed recall for ADP-LRP. Subjects with initial language symptoms performed lower on Boston naming test and categorical fluency (animals, vegetables) but better on logical memory delayed recall for the ADP and ADP-LRP groups.

For tests of visuospatial domain, there were a limited number of participants who completed the Benton line drawing test, while Trails-A duration and Trails-A correct lines were significantly lower in ADP and LRP groups with initial visuospatial symptoms.

#### Effect of *APOE-ε4* dose (1, 2, or none) on the model results

The ADP versus LRP logistic regression model using *APOE-ε4* dose was not feasible due to the small numbers of APOE*-ε4*/4 in LRP (*n* = 2). In a supplementary analysis comparing logistic regression models with (*APOE-ε4* present/absent) and *APOE-ε4* dose, we found that using the 3-level *APOE-ε4* does not improve the fit significantly for each of the initial cognitive symptoms (amnestic, executive, language, and visuospatial) compared to the 2-level *APOE-ε4* for ADP versus ADP-LRP. Details are provided in supplementary material.

#### Additional neuropathology evaluations

The distribution of initial visit Hachinski score versus the presence of any vascular pathology at autopsy is provided as supplementary material. Additionally, as the likelihood of having a DLB clinical syndrome is often thought to be lower among those with brainstem-predominant Lewy body pathology, in supplementary analyses when these cases were excluded from the LRP group (revised *n* = 65 from original *n* = 90) with less strict delineation of Lewy body pathology, the unadjusted model was still consistent with the prior results (analyses not presented).

## Discussion

These results from a well-characterized national neuropathology cohort point to a differential association between *APOE* ε4 genotype and initial cognitive symptoms among the ADP, LRP, and ADP-LRP groups. Consistent with our initial hypothesis one, *APOE* ε4 genotype was associated with an increased odds of initial amnestic symptoms compared to non-amnestic symptoms among the ADP and ADP-LRP pathology groups. Although the odds of amnestic symptoms in LRP did not reach statistical significance among *APOE* ε4 carriers, it also did not significantly differ from ADP in the adjusted model. There was, therefore, not enough evidence to conclude that ADP and LRP groups differ on the relationship between *APOE* ε4 and initial amnestic symptoms. Younger age was found to be consistently related to non-amnestic initial symptoms across all three groups, but contrary to our initial hypothesis, *APOE* ε4 carrier status impact on specific initial non-amnestic symptoms (language, visuospatial but not executive) differed by the nature of underlying neuropathology with commonalities between ADP and ADP-LRP groups but differing from the LRP group.

*APOE* ε4 genotype-related differences on neuroimaging within the medial temporal cortex have been extensively investigated given the close correspondence with initial AD symptoms of episodic memory loss ([29], review). In evaluating the underlying biology, *APOE* ε 4’s association with an increase in the aggregation and decrease in the clearance of Aβ has been well documented [30, 31]. A localized vulnerability with impairment of GABAergic interneurons in the hippocampus by *APOE* ε4, leading to learning and memory deficits among mice models, has also been reported [32]. ApoE protein accumulation in synapses and exacerbated synapse loss in human post-mortem brain tissue among *APOE* ε4 carriers is known [33, 34]. Along with synapse loss being a close correlate of cognitive changes in AD [35], these results taken together have been among the explanations for *APOE* ε 4 genotype’s correlation with the amnestic syndrome in early AD.

*APOE* ε4 genotype is also associated with increased risk of synucleinopathies [13] and with tau related neurodegeneration in animal models [36]. Even as a sequence of cortical involvement has been noted in DLB neuropathology corresponding to cognitive progression [17, 18], studies have so far been limited in evaluating if there is a differential effect of *APOE* ε4 genotype among initial cognitive symptoms related to LRP among earlier stage clinical subjects. Our results within neuropathology-characterized LRP and ADP-LRP clinical groups suggest that *APOE* ε4 genotype’s propensity towards amnestic symptoms is seen in the presence of both ADP and ADP-LRP neuropathology. In addition, *APOE* ε4 increased the odds of predominant visuospatial symptoms among LRP. Strikingly, given the prior result, the odds of amnestic initial symptoms in LRP did not reach statistical significance among *APOE* ε4 carriers, suggesting that any dependence between amnestic initial symptoms and LRP is likely weak in this group. These results need corroboration in a larger sample of LRP in future studies. Further, the dissimilar odds of non-amnestic symptoms among the LRP and ADP-LRP groups suggest that the impact of *APOE* ε 4 on initial symptoms could vary depending on the underlying degree of ADP and LRP pathology.

Our results in ADP parallel prior reports that *APOE ε4* carrier proportion was not elevated among aphasic variants of AD [10]. Among ADP with initial language symptoms in this cohort, even as they may not have been always related to aphasic variants, the strikingly lower proportion of *APOE* ε4 carriers among them (with worse Boston naming and verbal fluency scores) suggest that factors including female sex and environmental influences including higher education may play a role in language-related AD symptoms.

In reevaluating the hypothesis of *APOE ε4* being an anatomically selective risk factor that increases vulnerability to AD pathology in medial temporal regions alone [10], the current results noting higher odds of visuospatial initial symptoms among LRP *APOE ε4* carriers points to parietotemporal regions also as potential brain regions of vulnerability to *APOE ε4*’s effect. Interestingly, in PET studies among asymptomatic *APOE ε4* carriers, the largest correlation between the cerebral metabolic rate for glucose and *APOE ε4* status was noted in the parietotemporal regions of the brain [37]. The parietotemporal cortex was noted as having the highest degree of tau accumulation on ^8^F-flortaucipir PET among *APOE ε4* carriers compared to non-carriers regardless of the amyloid positive status [38]. Even though the specific mechanism of this parietotemporal vulnerability is unclear, one could speculate that regional neuronal energy metabolism vulnerabilities not unlike the posterior cingulate in AD are possible leading to early regional synaptic loss [39].

### Limitations and strengths

The use of initial cognitive symptoms as a window into understanding differential effects of *APOE* ε4 across different neuropathology is limited by the clinical subjectivity of documentation of initial cognitive symptoms. While not a substitute for a formal diagnosis of atypical AD syndromes as in the IWG-2 criteria [1], the large numbers of subjects included and the robustness of these effects even after taking into age, sex, and education as covariates point to strong trends that suggest differential impact of *APOE* ε4 on specific cognitive symptoms. It is therefore worthy of more detailed future studies to understand the biology of relative cognitive vulnerabilities detected. However, it is interesting to note that on average, among both the ADP and AD-LRP groups, those with amnestic initial symptoms performed significantly worse than those with non-amnestic initial symptoms on logical memory delayed recall but not on logical memory immediate recall (both verbal tests). This suggests that initial amnestic symptoms likely corresponded in these two groups for the most part with a hippocampal-dependent process [40, 41]. In contrast in the LRP group, those with amnestic initial symptoms did not differ significantly on logical memory immediate recall and delayed recall from the non-amnestic group suggesting that the initial amnestic symptoms in this specific group likely did not always correspond to a hippocampal-dependent process [40].

Antemortem hippocampal volumes in DLB have been reported to relate to the severity of neurofibrillary tangle pathology [42]. Prior studies among both AD (*APOE* ε 4/4 carriers, *n* = 34) and DLB (*APOE* ε 4/4 carriers, *n* = 7) patients noted that smaller hippocampal volumes on MRI were noted with an increasing burden of *APOE ε4* dosage [43], similar to that seen for cognitively normal middle-aged subjects [44]. Given the small number of *APOE* ε 4/4 carriers with LRP (*n* = 2) in our study, we have not been able to effectively evaluate the dosage effect of ε 4 allele on increasing odds of the amnestic syndrome in LRP. In our secondary analysis, even among ADP-LRP *APOE* ε 4/4 carriers (*n* = 142), we report that accounting for the dosage of ε 4 alleles (0, 1, or 2) did not improve the fit of our logistical models predicting amnestic or non-amnestic symptoms when comparing ADP and ADP-LRP. This suggests that the odds of initial amnestic symptoms is not significantly different even after taking *APOE* ε 4/4 status into account among those with underlying ADP or ADP-LRP pathology. Future studies directly evaluating MRI hippocampal volumes among a larger number of subjects are needed to clarify if *APOE* ε 4/4 status additionally impacts hippocampal volume in a clinically significant manner when specific ADP and LRP pathology is present.

CDR-G ≤ 1 was used to limit analysis to participants at the initial visit who were more likely to provide an accurate history of initial cognitive symptoms from recent history. Use of CDR-G score to determine subjects with early stages of dementia or mild cognitive impairment also has its limitations, as some domains (language, behavior) are not well captured by the standard CDR-G, and participants could be much further along in the disease course potentially limiting the accuracy of their history of initial cognitive symptoms like language. Despite the large size of the initial NACC cohort, sub-stratification by initial cognitive symptoms also makes the analysis of some subgroups small. This limits our analysis on questions of interest to this study, especially relating to LRP given the low number of subjects with some neuropsychology tests. Similar analysis after sub stratification by ε3 and ε2 also decreases the power of the analysis given the smaller number of ε3/ε2 and ε3/ε2s. Further, it is less likely that any protective effect of ε2 genotypes to AD onset specifically increases the odds of initial amnestic symptoms among ε4 carriers over non-amnestic symptoms in the statistical model among autopsy-confirmed AD subjects. Additionally, given the prevalence of mixed dementia from vascular etiology, we evaluated the Hachinski ischemic scale scores across all groups were < 2, which is well below the threshold for multi-infarct dementia pathology discrimination from AD at a score of ≤ 4 [45]. This could be taken as supportive of relatively little contribution from multi-infarcts to the results on initial cognitive symptoms. Hachinski score at initial visit rather than neuropathology of vascular disease burden at autopsy was considered in this study, as the vascular burden could potentially change from initial visit to autopsy (e.g., stroke) for some subjects limiting their utility in evaluating their effect on symptoms at the initial visit. Furthermore, in NACC, the presence of one or more ischemic, hemorrhagic, or vascular pathology (including mild severity indicated for pathologies such as atherosclerosis) is documented as present, absent, or unknown to standardize the characterization of vascular pathology across centers. This makes it difficult to accurately find correlation values between antemortem Hachinski score at the initial visit and categorical variable of presence versus absence of vascular pathology at autopsy.

*APOE* ε4 carrier rates can vary from study to study given the biases in participant population recruitment impacting the odds ratio; this is mitigated to a degree by the number of subjects in the amnestic versus non-amnestic group analysis. ADCs are focused on Alzheimer’s disease and related dementias, including Lewy body dementia, but recruitment practices by cognitive status and disease etiology may vary by ADC impacting the number of LRP participants and the degree of their neuropathology characterization. The number of ADP-LRP frequency is on the higher side (895 of a total of 985 cases with LRP, 90.8%) in this multi-center study compared to prior single-center studies which were often below 75% [46, 47]. Probable reasons to consider include (a) the data in this National Alzheimer’s Disease Center database likely had a bias towards AD clinical cases especially in the early years of the program; (b) inclusion of young-onset cases in this dataset (avg 73.79 years, std. dev 10.7 at first visit for ADP-LRP) does not exclude coexisting LRP [48]; (c) it is also likely that with the improvement of staining practices for LRP which have been standardized, LRP may have been more likely to be detected than in prior reports [49].

Another issue is missing data; this was addressed by limiting analysis to participants with completed data fields in the key variables of interest, and given the key differences in cognitive domains across different pathologies by *APOEε4* status being the primary hypothesis, data imputation of cognitive data or missing *APOEε4* data were not considered most appropriate in this context. Visual hallucinations and non-cognitive symptoms, though are striking features of DLB, were not investigated in this current report given the limitations of data available pertinent to a detailed analysis with the framework of this study. The study’s strength is the evaluation of the etiology of underlying dementia following neuropathology evaluation in addition to the initial clinical symptoms for some key well-powered results. Given the strengths and biases of the NACC cohort, it is likely the current results are generalizable to other prospective research cohorts tracking *APOE* ε4 carriers and non-carriers including clinical trials.

## Conclusions

Our results clearly show that the odds of amnestic and non-amnestic initial symptoms with *APOE* ε4 varies with underlying neuropathology. *APOE* ε4 when present with AD neuropathology by itself or with co-existing LRP is more likely to present with amnestic symptoms and lowering the odds of language-related initial symptoms. These results also suggest *APOE* ε4 likely increases the odds of visuospatial initial symptoms with LRP. These results of initial cognitive symptom propensities suggest distinct interaction between regional brain effects of *APOE* ε4 and the underlying neuropathology. These results raise the need for future studies to evaluate if there are any underlying true biologic interactions between *APOE* ε4 gene and neuropathology impacting specific neural sub-networks.

## Supplementary Information

**Additional file 1: Supplementary Table 1.** Clinical and neurocognitive data from Alzheimer’s neuropathology group for amnestic, executive/−attention concentration, language and visuospatial initial symptoms.

**Additional file 2: Supplementary Table 2.** Clinical and neurocognitive data from Lewy body neuropathology group for amnestic, executive/−attention concentration, language and visuospatial initial symptoms.

**Additional file 3: Supplementary Table 3.** Clinical and neurocognitive data from the mixed Lewy body-Alzheimer’s neuropathology group for amnestic, executive/−attention concentration, language and visuospatial initial symptoms.

**Additional file 4: Supplementary Table 4.** Coefficients of all logistic regression models.

**Additional file 5: Supplementary material.**

## Data Availability

The datasets analyzed during the current study are available in the NACC repository, https://www.alz.washington.edu.
