# Supplementary material for: Impact of APOE ε4 genotype on initial cognitive symptoms differs for Alzheimer’s and Lewy body neuropathology
Source: Alzheimers Res Ther. 2021 Jan 23;13:31. doi: 10.1186/s13195-021-00771-1 (PMC7825215; doi:10.1186/s13195-021-00771-1)
Supplement: Supplementary file 1 — Additional file 1: Supplementary Table 1. Clinical and neurocognitive data from Alzheimer’s neuropathology group for amnestic, executive/−attention concentration, language and visuospatial initial symptoms. [file 13195_2021_771_MOESM1_ESM.docx]

|  | Non-amnestic initial symptom | | | Amnestic initial symptom | | |  |  |  |
| --- | --- | --- | --- | --- | --- | --- | --- | --- | --- |
|  | **N** | **Mean** | **Std. Deviation** | **N** | **Mean** | **Std. Deviation** | **F** | **Sig.** | **Eta Squared** |
| Age at visit | 254 | 68.6693 | 10.48361 | 1043 | 78.2531 | 9.72171 | 192.382 | **<0.0001** | **0.129** |
| Sex F% | 46.1% | | | 45.6% | | | 0.015 | 0.9 |  |
| EDUCATION | 251 | 15.76 | 2.862 | 1039 | 15.23 | 3.101 | 6.128 | 0.013 | 0.005 |
| APOEε4% | 47.2% | | | 53.9% | | | 3.72 | 0.054 | 0 |
| Hachinski score | 247 | 0.98 | 1.56 | 1025 | 0.98 | 1.35 | 0.002 | 0.96 |  |
| MMSE | 243 | 22.46 | 5.926 | 1012 | 23.51 | 4.352 | 9.863 | 0.002 | 0.008 |
| LOGICAL MEMORY immediate | 226 | 6.07 | 4.738 | 962 | 5.64 | 4.189 | 1.78 | 0.182 | 0.001 |
| LOGICAL MEMORY delayed | 226 | 4.88 | 4.885 | 964 | 3.14 | 4.101 | 30.78 | **<0.0001** | **0.025** |
| DIGIT SPAN FORWARD LENGTH | 235 | 5.36 | 1.55 | 984 | 6.13 | 1.223 | 67.091 | **<0.0001** | **0.052** |
| DIGIT SPAN BACKWARD LENGTH | 230 | 3.27 | 1.273 | 983 | 3.95 | 1.171 | 61.435 | **<0.0001** | **0.048** |
| ANIMALS 60sec | 241 | 10.55 | 5.371 | 999 | 12.31 | 5.277 | 21.432 | **<0.0001** | **0.017** |
| VEGETABLES 60sec | 230 | 6.67 | 4.29 | 983 | 7.93 | 3.845 | 19.012 | **<0.0001** | **0.015** |
| TRAIL A Seconds | 225 | 68.62 | 39.502 | 955 | 60.64 | 33.937 | 9.437 | 0.002 | 0.008 |
| TRAIL A CORRECT LINES | 135 | 22.36 | 4.996 | 331 | 23.41 | 2.946 | 7.914 | 0.005 | 0.017 |
| TRAIL B Seconds | 180 | 198.88 | 84.836 | 815 | 185.59 | 86.453 | 3.509 | 0.061 | 0.004 |
| TRAIL B  CORRECT LINES | 104 | 20.14 | 6.478 | 278 | 20.54 | 6.512 | 0.28 | 0.597 | 0.001 |
| WAIS-R digit symbol | 203 | 27.21 | 13.312 | 866 | 29.62 | 12.64 | 5.832 | 0.016 | 0.005 |
| BOSTON Naming test | 229 | 20.73 | 7.765 | 965 | 22.31 | 5.946 | 11.475 | 0.001 | 0.01 |
| Eta squared > 0.1 and p value<0.05 are in bold | | | | | | | | | |

**Supplementary Table 1:** Clinical and neurocognitive data from Alzheimer’s neuropathology group for amnestic, executive/-attention concentration, language and visual spatial initial symptoms.

|  | Non-Executive/Attention initial symptom | | | Executive/Attention initial symptom | | |  |  |  |
| --- | --- | --- | --- | --- | --- | --- | --- | --- | --- |
|  | **N** | **Mean** | **Std. Deviation** | **N** | **Mean** | **Std. Deviation** | **F** | **Sig.** | **Eta Squared** |
| Age at visit | 1221 | 76.8526 | 10.27473 | 76 | 68.7237 | 12.39634 | 43.632 | **<0.0001** | **0.033** |
| Sex F% | 46.1% | | | 38.2% | | | 1.86 | 0.17 |  |
| EDUCATION | 1215 | 15.32 | 3.059 | 75 | 15.53 | 3.116 | 0.329 | 0.566 | 0 |
| APOEε4% | 52.7% | | | 52.6% | | | 0 | 1 |  |
| Hachinski score | 1198 | 0.99 | 1.39 | 74 | 0.84 | 1.47 | 0.81 | 0.37 | 0.001 |
| MMSE | 1181 | 23.3 | 4.688 | 74 | 23.54 | 5.145 | 0.187 | 0.666 | 0 |
| LOGICAL MEMORY immediate | 1118 | 5.7 | 4.272 | 70 | 6.14 | 4.737 | 0.709 | 0.4 | 0.001 |
| LOGICAL MEMORY delayed | 1120 | 3.41 | 4.276 | 70 | 4.39 | 4.822 | 3.36 | 0.067 | 0.003 |
| DIGIT SPAN FORWARD LENGTH | 1145 | 6 | 1.328 | 74 | 5.74 | 1.283 | 2.554 | 0.11 | 0.002 |
| DIGIT SPAN BACKWARD LENGTH | 1140 | 3.86 | 1.206 | 73 | 3.23 | 1.297 | 18.469 | **<0.0001** | **0.015** |
| ANIMALS 60sec | 1166 | 11.99 | 5.347 | 74 | 11.66 | 5.248 | 0.26 | 0.61 | 0 |
| VEGETABLES 60sec | 1139 | 7.73 | 3.969 | 74 | 7.04 | 3.819 | 2.103 | 0.147 | 0.002 |
| TRAIL A Seconds | 1110 | 61.84 | 34.753 | 70 | 67.17 | 41.464 | 1.511 | 0.219 | 0.001 |
| TRAIL A CORRECT LINES | 424 | 23.22 | 3.476 | 42 | 21.95 | 5.277 | 4.585 | 0.033 | 0.01 |
| TRAIL B Seconds | 939 | 187.79 | 86.154 | 56 | 191.3 | 88.967 | 0.087 | 0.767 | 0 |
| TRAIL B  CORRECT LINES | 351 | 20.43 | 6.526 | 31 | 20.42 | 6.265 | 0 | 0.991 | 0 |
| WAIS-R digit symbol | 1006 | 29.25 | 12.8 | 63 | 27.73 | 12.786 | 0.835 | 0.361 | 0.001 |
| BOSTON Naming test | 1121 | 21.93 | 6.392 | 73 | 23.27 | 5.777 | 3.082 | 0.079 | 0.003 |
| Eta squared > 0.1 and p value<0.05 are in bold | | | | | | | | | |

|  | Non-language initial symptom | | | Language initial symptom | | |  |  |  |
| --- | --- | --- | --- | --- | --- | --- | --- | --- | --- |
|  | **N** | **Mean** | **Std. Deviation** | **N** | **Mean** | **Std. Deviation** | **F** | **Sig.** | **Eta Squared** |
| Age at visit | 1148 | 77.2814 | 10.36358 | 149 | 69.4027 | 9.61061 | 77.461 | **<0.0001** | **0.056** |
| Sex F% | 45% | | | 51% | | | 1.9 | 0.17 |  |
| EDUCATION | 1143 | 15.27 | 3.086 | 147 | 15.88 | 2.818 | 5.331 | 0.021 | 0.004 |
| APOEε4% | 53.4% | | | 47% | | | 2.1 | 0.14 |  |
| Hachinski score | 1126 | 0.99 | 1.39 | 146 | 0.92 | 1.47 | 0.32 | 0.57 | 0 |
| MMSE | 1112 | 23.5 | 4.414 | 143 | 21.83 | 6.428 | 16.223 | **<0.0001** | **0.013** |
| LOGICAL MEMORY immediate | 1056 | 5.7 | 4.239 | 132 | 5.89 | 4.772 | 0.234 | 0.628 | 0 |
| LOGICAL MEMORY delayed | 1058 | 3.28 | 4.198 | 132 | 5.02 | 4.898 | 19.543 | **<0.0001** | **0.016** |
| DIGIT SPAN FORWARD LENGTH | 1084 | 6.1 | 1.228 | 135 | 5.04 | 1.672 | 82.15 | **<0.0001** | **0.063** |
| DIGIT SPAN BACKWARD LENGTH | 1081 | 3.89 | 1.192 | 132 | 3.26 | 1.305 | 32.709 | **<0.0001** | **0.026** |
| ANIMALS 60sec | 1102 | 12.25 | 5.274 | 138 | 9.74 | 5.352 | 27.676 | **<0.0001** | **0.022** |
| VEGETABLES 60sec | 1084 | 7.87 | 3.868 | 129 | 6.15 | 4.404 | 22.194 | **<0.0001** | **0.018** |
| TRAIL A Seconds | 1048 | 62.12 | 35.47 | 132 | 62.42 | 33.003 | 0.008 | 0.929 | 0 |
| TRAIL A CORRECT LINES | 386 | 23.13 | 3.501 | 80 | 22.99 | 4.493 | 0.106 | 0.745 | 0 |
| TRAIL B Seconds | 886 | 186.72 | 86.479 | 109 | 198.33 | 84.252 | 1.76 | 0.185 | 0.002 |
| TRAIL B  CORRECT LINES | 318 | 20.39 | 6.572 | 64 | 20.64 | 6.152 | 0.079 | 0.779 | 0 |
| WAIS-R digit symbol | 944 | 29.31 | 12.718 | 125 | 28.06 | 13.39 | 1.053 | 0.305 | 0.001 |
| BOSTON Naming test | 1062 | 22.42 | 5.932 | 132 | 18.72 | 8.447 | 40.982 | **<0.0001** | **0.033** |
| Eta squared > 0.1 and p value<0.05 are in bold | | | | | | | | | |

|  | Non-visuospatial initial symptom | | | Visuospatial initial symptom | | |  |  |  |
| --- | --- | --- | --- | --- | --- | --- | --- | --- | --- |
|  | **N** | **Mean** | **Std. Deviation** | **N** | **Mean** | **Std. Deviation** | **F** | **Sig.** | **Eta Squared** |
| Age at visit | 1268 | 76.642 | 10.47255 | 29 | 64.7586 | 8.63405 | 36.759 | **<0.0001** | **0.028** |
| Sex F% | 45.8% | | | 41.3% | | | 0.225 | 0.63 |  |
| EDUCATION | 1261 | 15.33 | 3.075 | 29 | 15.76 | 2.415 | 0.564 | 0.453 | 0 |
| APOEε4% | 53.1% | | | 34.5% | | | 3.9 | **0.047** |  |
| Hachinski score | 1245 | 0.96 | 1.38 | 27 | 1.67 | 2.09 | 6.7 | 0.01 | 0.005 |
| MMSE | 1229 | 23.32 | 4.716 | 26 | 22.88 | 4.693 | 0.217 | 0.642 | 0 |
| LOGICAL MEMORY immediate | 1164 | 5.7 | 4.291 | 24 | 6.79 | 4.681 | 1.513 | 0.219 | 0.001 |
| LOGICAL MEMORY delayed | 1166 | 3.43 | 4.288 | 24 | 5.58 | 5.064 | 5.905 | 0.015 | 0.005 |
| DIGIT SPAN FORWARD LENGTH | 1193 | 5.98 | 1.331 | 26 | 5.96 | 1.148 | 0.006 | 0.937 | 0 |
| DIGIT SPAN BACKWARD LENGTH | 1188 | 3.83 | 1.223 | 25 | 3.44 | 1.044 | 2.526 | 0.112 | 0.002 |
| ANIMALS 60sec | 1211 | 11.98 | 5.343 | 29 | 11.59 | 5.261 | 0.153 | 0.696 | 0 |
| VEGETABLES 60sec | 1186 | 7.68 | 3.947 | 27 | 8.15 | 4.655 | 0.373 | 0.542 | 0 |
| TRAIL A Seconds | 1157 | 61.23 | 34.339 | 23 | 108.61 | 45.834 | 42.307 | **<0.0001** | **0.035** |
| TRAIL A CORRECT LINES | 453 | 23.2 | 3.547 | 13 | 19.85 | 6.375 | 10.704 | **0.001** | **0.023** |
| TRAIL B Seconds | 980 | 187.33 | 86.366 | 15 | 231.13 | 69.435 | 3.819 | 0.051 | 0.004 |
| TRAIL B  CORRECT LINES | 373 | 20.55 | 6.415 | 9 | 15.67 | 8.382 | 5.011 | **0.026** | **0.013** |
| WAIS-R digit symbol | 1054 | 29.32 | 12.743 | 15 | 18 | 12.095 | 11.683 | **0.001** | **0.011** |
| BOSTON Naming test | 1170 | 21.97 | 6.372 | 24 | 24.08 | 5.602 | 2.609 | 0.107 | 0.002 |
| Eta squared > 0.1 and p value<0.05 are in bold | | | | | | | | | |
