# Supplementary material for: Impact of APOE ε4 genotype on initial cognitive symptoms differs for Alzheimer’s and Lewy body neuropathology
Source: Alzheimers Res Ther. 2021 Jan 23;13:31. doi: 10.1186/s13195-021-00771-1 (PMC7825215; doi:10.1186/s13195-021-00771-1)
Supplement: Supplementary file 2 — Additional file 2: Supplementary Table 2. Clinical and neurocognitive data from Lewy body neuropathology group for amnestic, executive/−attention concentration, language and visuospatial initial symptoms. [file 13195_2021_771_MOESM2_ESM.docx]

|  | Non-amnestic initial symptom | | | Amnestic initial symptom | | |  |
| --- | --- | --- | --- | --- | --- | --- | --- |
|  | **N** | **Mean** | **Std. Deviation** | **N** | **Mean** | **Std. Deviation** | **Sig.** |
| Age at visit | 37 | 68.5135 | 7.87267 | 52 | 76.5 | 8.76378 | **<0.0001** |
| Sex %F | 37 | 37.8% | | 52 | 17.3% | | **0.029** |
| EDUCATION | 37 | 15.54 | 3.313 | 52 | 15.65 | 2.883 | 0.1 |
| APOEε4% | 37 | 24.3% | | 52 | 17.3% | | 0.42 |
| Hachinski score | 36 | 1.0 | 1.7 | 51 | 1.14 | 1.76 |  |
| MMSE | 35 | 23.69 | 5.028 | 50 | 25.2 | 3.362 | 0.26 |
| LOGICAL MEMORY immediate | 31 | 7.06 | 4.633 | 48 | 8.35 | 4.315 | 0.23 |
| LOGICAL MEMORY delayed | 31 | 6.71 | 4.421 | 48 | 6.02 | 4.102 | 0.53 |
| DIGIT SPAN FORWARD LENGTH | 32 | 5.56 | 1.366 | 49 | 6.33 | 1.162 | **0.01** |
| DIGIT SPAN BACKWARD LENGTH | 32 | 3.47 | 1.244 | 49 | 3.67 | 0.966 | 0.42 |
| ANIMALS 60sec | 34 | 9.74 | 5.395 | 50 | 13.18 | 5.879 | **0.01** |
| VEGETABLES 60sec | 33 | 7.06 | 4.046 | 49 | 8.16 | 3.294 | 0.16 |
| TRAIL A Seconds | 30 | 79.7 | 43.693 | 50 | 76.24 | 38.542 | 0.89 |
| TRAIL A CORRECT LINES | 15 | 23.13 | 1.959 | 17 | 23.12 | 2.781 | 0.74 |
| TRAIL B Seconds | 23 | 193.83 | 85.568 | 39 | 203.13 | 88.143 | 0.84 |
| TRAIL B  CORRECT LINES | 12 | 20.25 | 6.21 | 12 | 20.75 | 7.3 | 0.76 |
| WAIS-R digit symbol | 28 | 27.61 | 16.344 | 44 | 29.3 | 12.094 | 0.6 |
| BOSTON Naming test | 31 | 22.03 | 8.849 | 49 | 25.45 | 4.444 | 0.16 |
|  | p value<0.05 are in bold, Mann Whitney tests | | | | | |  |

**Supplementary Table 2:** Clinical and neurocognitive data from Lewy body neuropathology group for amnestic, executive/-attention concentration, language and visual spatial initial symptoms.

|  | Non-Executive initial symptom | | | Executive/Attention initial symptom | | |  |
| --- | --- | --- | --- | --- | --- | --- | --- |
|  | **N** | **Mean** | **Std. Deviation** | **N** | **Mean** | **Std. Deviation** | **Sig.** |
| Age at visit | 79 | 73.5949 | 9.18265 | 10 | 69.9 | 9.59687 | 0.26 |
| Sex %F | 79 | 26.6% | | 10 | 20% | | 0.65 |
| EDUCATION | 79 | 15.61 | 2.998 | 10 | 15.6 | 3.627 | 0.78 |
| APOEε4% | 79 | 21.5% | | 10 | 10% | | 0.39 |
| Hachinski score | 77 | 1.05 | 1.74 | 10 | 1.3 | 1.7 |  |
| MMSE | 75 | 24.65 | 4.115 | 10 | 24 | 4.761 | 0.79 |
| LOGICAL MEMORY immediate | 69 | 7.88 | 4.542 | 10 | 7.6 | 4.033 | 0.98 |
| LOGICAL MEMORY delayed | 69 | 6.14 | 4.233 | 10 | 7.3 | 4.165 | 0.45 |
| DIGIT SPAN FORWARD LENGTH | 71 | 6.07 | 1.269 | 10 | 5.7 | 1.494 | 0.49 |
| DIGIT SPAN BACKWARD LENGTH | 71 | 3.69 | 1.022 | 10 | 2.9 | 1.287 | 0.09 |
| ANIMALS 60sec | 74 | 11.77 | 6.049 | 10 | 11.9 | 4.977 | 0.88 |
| VEGETABLES 60sec | 72 | 7.65 | 3.674 | 10 | 8.2 | 3.458 | 0.71 |
| TRAIL A Seconds | 71 | 77.85 | 39.749 | 9 | 75.11 | 47.054 | 0.52 |
| TRAIL A CORRECT LINES | 28 | 23 | 2.539 | 4 | 24 | 0 | 0.6 |
| TRAIL B Seconds | 55 | 199.4 | 86.196 | 7 | 201.86 | 96.889 | 0.91 |
| TRAIL B  CORRECT LINES | 20 | 21.15 | 6.784 | 4 | 17.25 | 5.377 | 0.14 |
| WAIS-R digit symbol | 63 | 28.97 | 13.056 | 9 | 26.33 | 19.131 | 0.66 |
| BOSTON Naming test | 70 | 23.9 | 6.981 | 10 | 25.7 | 3.773 | 0.61 |
| p value<0.05 are in bold, Mann Whitney tests | | | | | | | |

|  | Non-language initial symptom | | | Language initial symptom | | |  |
| --- | --- | --- | --- | --- | --- | --- | --- |
|  | **N** | **Mean** | **Std. Deviation** | **N** | **Mean** | **Std. Deviation** | **Sig.** |
| Age at visit | 71 | 74.9155 | 9.05183 | 18 | 66.3333 | 6.57088 | <**0.0001** |
| Sex %F | 71 | 18.3% | | 18 | 55.6% | | **0.001** |
| EDUCATION | 71 | 15.62 | 2.997 | 18 | 15.56 | 3.347 | 0.93 |
| APOEε4% | 71 | 23.9% | | 18 | 5.6% | | 0.083 |
| Hachinski score | 70 | 1.17 | 1.84 | 17 | 0.71 | 1.11 |  |
| MMSE | 69 | 25.12 | 3.517 | 16 | 22.25 | 5.848 | 0.07 |
| LOGICAL MEMORY immediate | 67 | 8.18 | 4.348 | 12 | 6 | 4.805 | 0.1 |
| LOGICAL MEMORY delayed | 67 | 6.28 | 4.074 | 12 | 6.33 | 5.14 | 0.88 |
| DIGIT SPAN FORWARD LENGTH | 68 | 6.16 | 1.205 | 13 | 5.31 | 1.548 | **0.03** |
| DIGIT SPAN BACKWARD LENGTH | 68 | 3.63 | 1.078 | 13 | 3.38 | 1.121 | 0.37 |
| ANIMALS 60sec | 69 | 12.62 | 5.688 | 15 | 7.93 | 5.496 | **0.005** |
| VEGETABLES 60sec | 68 | 8.16 | 3.304 | 14 | 5.57 | 4.467 | **0.02** |
| TRAIL A Seconds | 66 | 77.62 | 39.02 | 14 | 77.14 | 47.559 | 0.56 |
| TRAIL A CORRECT LINES | 24 | 23.38 | 2.356 | 8 | 22.38 | 2.504 | 0.25 |
| TRAIL B Seconds | 52 | 206.42 | 87.041 | 10 | 164.6 | 79.238 | 0.19 |
| TRAIL B  CORRECT LINES | 18 | 20.33 | 6.598 | 6 | 21 | 7.348 | 0.67 |
| WAIS-R digit symbol | 59 | 28.22 | 13.28 | 13 | 30.54 | 16.496 | 0.78 |
| BOSTON Naming test | 68 | 25.56 | 4.314 | 12 | 16 | 11.037 | **0.001** |
| p value<0.05 are in bold, Mann Whitney tests | | | | | | | |

|  | Non-visuospatial initial symptom | | | Visuospatial initial symptom | | |  |
| --- | --- | --- | --- | --- | --- | --- | --- |
|  | **N** | **Mean** | **Std. Deviation** | **N** | **Mean** | **Std. Deviation** | **Sig.** |
| Age at visit | 80 | 73.3875 | 9.41382 | 9 | 71.3333 | 7.84219 | 0.6 |
| Sex %F | 80 | 26.3% | | 9 | 22.2% | | 0.79 |
| EDUCATION | 80 | 15.62 | 3.046 | 9 | 15.44 | 3.283 | 0.68 |
| APOEε4% | 80 | 13.8% | | 9 | 77.8% | | **<0.001** |
| Hachinski score | 78 | 1.06 | 1.62 | 9 | 1.22 | 2.59 |  |
| MMSE | 76 | 24.42 | 4.287 | 9 | 25.89 | 2.848 | 0.45 |
| LOGICAL MEMORY immediate | 70 | 7.84 | 4.389 | 9 | 7.89 | 5.255 | 0.96 |
| LOGICAL MEMORY delayed | 70 | 6.26 | 4.259 | 9 | 6.56 | 4.096 | 0.73 |
| DIGIT SPAN FORWARD LENGTH | 72 | 6.06 | 1.331 | 9 | 5.78 | 0.972 | 0.44 |
| DIGIT SPAN BACKWARD LENGTH | 72 | 3.51 | 1.061 | 9 | 4.22 | 1.093 | 0.12 |
| ANIMALS 60sec | 75 | 11.96 | 5.992 | 9 | 10.33 | 5.196 | 0.45 |
| VEGETABLES 60sec | 73 | 7.67 | 3.659 | 9 | 8.11 | 3.586 | 0.86 |
| TRAIL A Seconds | 73 | 76.27 | 40.812 | 7 | 90.71 | 34.437 | 0.21 |
| TRAIL A CORRECT LINES | 29 | 23.03 | 2.5 | 3 | 24 | 0 | 0.67 |
| TRAIL B Seconds | 56 | 196.09 | 87.402 | 6 | 233.17 | 77.419 | 0.27 |
| TRAIL B  CORRECT LINES | 22 | 20.18 | 6.85 | 2 | 24 | - | - |
| WAIS-R digit symbol | 66 | 29.14 | 13.912 | 6 | 23.17 | 12.449 | 0.41 |
| BOSTON Naming test | 71 | 23.89 | 6.882 | 9 | 26 | 4.583 | 0.32 |
| p value<0.05 are in bold, Mann Whitney tests | | | | | | | |
