# Supplementary material for: Impact of APOE ε4 genotype on initial cognitive symptoms differs for Alzheimer’s and Lewy body neuropathology
Source: Alzheimers Res Ther. 2021 Jan 23;13:31. doi: 10.1186/s13195-021-00771-1 (PMC7825215; doi:10.1186/s13195-021-00771-1)
Supplement: Supplementary file 3 — Additional file 3: Supplementary Table 3. Clinical and neurocognitive data from the mixed Lewy body-Alzheimer’s neuropathology group for amnestic, executive/−attention concentration, language and visuospatial initial symptoms. [file 13195_2021_771_MOESM3_ESM.docx]

|  | Non-amnestic initial symptom | | | Amnestic initial symptom | | |  |  |  |
| --- | --- | --- | --- | --- | --- | --- | --- | --- | --- |
|  | **N** | **Mean** | **Std. Deviation** | **N** | **Mean** | **Std. Deviation** | **F** | **Sig.** | **Eta Squared** |
| Age at visit | 168 | 70.0119 | 9.37316 | 722 | 74.687 | 10.01976 | 30.384 | **<0.0001** | **0.033** |
| Sex F% | 27.3% | | | 39.9% | | | **9.1** | **0.003** |  |
| EDUCATION | 166 | 16.19 | 2.646 | 718 | 15.34 | 3.154 | 10.357 | **0.001** | **0.012** |
| APOEε4% | 53% | | | 63.7% | | | **6.6** | **0.01** |  |
| Hachinski score | 163 | 0.8 | 1.36 | 704 | 0.83 | 1.23 | 0.06 | 0.8 | 0 |
| MMSE | 161 | 21.88 | 5.744 | 692 | 23.01 | 4.592 | 7.181 | 0.008 | 0.008 |
| LOGICAL MEMORY immediate | 149 | 5.44 | 4.324 | 663 | 4.96 | 3.917 | 1.745 | 0.187 | 0.002 |
| LOGICAL MEMORY delayed | 149 | 4.1 | 4.07 | 660 | 2.82 | 3.652 | 14.388 | **<0.0001** | **0.018** |
| DIGIT SPAN FORWARD LENGTH | 155 | 5.29 | 1.595 | 675 | 6.15 | 1.199 | 56.427 | **<0.0001** | **0.064** |
| DIGIT SPAN BACKWARD LENGTH | 150 | 3.12 | 1.247 | 672 | 3.91 | 1.234 | 50.56 | **<0.0001** | **0.058** |
| ANIMALS 60sec | 157 | 10.38 | 5.488 | 698 | 12.27 | 5.191 | 16.67 | **<0.0001** | **0.019** |
| VEGETABLES 60sec | 154 | 6.45 | 3.837 | 691 | 7.64 | 3.806 | 12.236 | **<0.0001** | **0.014** |
| TRAIL A Seconds | 147 | 87.84 | 44.599 | 667 | 65.26 | 38.451 | 39.11 | **<0.0001** | **0.046** |
| TRAIL A CORRECT LINES | 86 | 21.57 | 5.217 | 285 | 23.18 | 2.883 | 13.566 | **<0.0001** | **0.035** |
| TRAIL B Seconds | 111 | 235.23 | 84.027 | 555 | 196.67 | 90.354 | 17.231 | **<0.0001** | **0.025** |
| TRAIL B  CORRECT LINES | 61 | 17.8 | 7.582 | 234 | 19.82 | 7.233 | 3.704 | 0.055 | 0.012 |
| WAIS-R digit symbol | 132 | 21.39 | 12.971 | 599 | 27.01 | 13.464 | 19.139 | **<0.0001** | **0.026** |
| BOSTON Naming test | 149 | 20.37 | 7.609 | 669 | 22.47 | 6.191 | 12.853 | **<0.0001** | **0.016** |
| Eta squared > 0.1 and p value<0.05 are in bold | | | | | | | | |  |

**Supplementary Table 3:** Clinical and neurocognitive data from mixed Lewy body-Alzheimer’s neuropathology group for amnestic, executive/-attention concentration, language and visual spatial initial symptoms.

|  | Non-Executive/Attention initial symptom | | | Executive/Attention initial symptom | | |  |  |  |
| --- | --- | --- | --- | --- | --- | --- | --- | --- | --- |
|  | **N** | **Mean** | **Std. Deviation** | **N** | **Mean** | **Std. Deviation** | **F** | **Sig.** | **Eta Squared** |
| Age at visit | 832 | 74.0481 | 9.9445 | 58 | 70.3103 | 11.15946 | 7.534 | 0.006 | 0.008 |
| Sex F% | 38.4% | | | 24.1% | | | **4.7** | **0.029** |  |
| EDUCATION | 826 | 15.47 | 3.106 | 58 | 15.84 | 2.72 | 0.792 | 0.374 | 0.001 |
| APOEε4% | 61.7% | | | 60.3% | | | 0.047 | 1 |  |
| Hachinski score | 811 | 0.82 | 1.23 | 56 | 0.86 | 1.53 | 0.037 | 0.85 | 0 |
| MMSE | 796 | 22.71 | 4.884 | 57 | 24.02 | 4.155 | 3.862 | 0.05 | 0.005 |
| LOGICAL MEMORY immediate | 758 | 4.96 | 3.952 | 54 | 6.31 | 4.42 | 5.87 | 0.016 | 0.007 |
| LOGICAL MEMORY delayed | 755 | 2.93 | 3.676 | 54 | 4.76 | 4.522 | **12.058** | **0.001** | **0.015** |
| DIGIT SPAN FORWARD LENGTH | 774 | 5.99 | 1.325 | 56 | 5.93 | 1.319 | 0.121 | 0.728 | 0 |
| DIGIT SPAN BACKWARD LENGTH | 766 | 3.79 | 1.269 | 56 | 3.43 | 1.291 | 4.313 | 0.038 | 0.005 |
| ANIMALS 60sec | 798 | 11.97 | 5.294 | 57 | 11.16 | 5.291 | 1.267 | 0.261 | 0.001 |
| VEGETABLES 60sec | 787 | 7.45 | 3.867 | 58 | 7.09 | 3.409 | 0.488 | 0.485 | 0.001 |
| TRAIL A Seconds | 759 | 68 | 40.133 | 55 | 87.85 | 42.024 | **12.472** | **<0.0001** | **0.015** |
| TRAIL A CORRECT LINES | 340 | 22.88 | 3.504 | 31 | 22 | 4.698 | 1.692 | 0.194 | 0.005 |
| TRAIL B Seconds | 623 | 200.7 | 90.679 | 43 | 237.74 | 79.768 | 6.81 | **0.009** | **0.01** |
| TRAIL B  CORRECT LINES | 272 | 19.71 | 7.19 | 23 | 15.78 | 8.257 | **6.19** | **0.013** | **0.021** |
| WAIS-R digit symbol | 679 | 26.37 | 13.711 | 52 | 21.17 | 10.023 | **7.164** | **0.008** | **0.01** |
| BOSTON Naming test | 765 | 21.98 | 6.598 | 53 | 23.6 | 5.043 | 3.072 | 0.08 | 0.004 |
| Eta squared > 0.1 and p value<0.05 are in bold | | | | | | | | | |

|  | Non-language initial symptom | | | Language initial symptom | | |  |  |  |
| --- | --- | --- | --- | --- | --- | --- | --- | --- | --- |
|  | **N** | **Mean** | **Std. Deviation** | **N** | **Mean** | **Std. Deviation** | **F** | **Sig.** | **Eta Squared** |
| Age at visit | 808 | 74.1423 | 10.16717 | 82 | 70.4756 | 8.32552 | 9.983 | **0.002** | **0.011** |
| Sex F% | 38.6% | | | 26.8% | | | 4.4 | **0.036** |  |
| EDUCATION | 804 | 15.39 | 3.1 | 80 | 16.59 | 2.675 | 11.147 | **0.001** | **0.012** |
| APOEε4% | 63.2% | | | 46.3% | | | 9.0 | **0.003** |  |
| Hachinski score | 787 | 0.85 | 1.27 | 80 | 0.59 | 1.09 | 3.2 | 0.074 | 0.004 |
| MMSE | 775 | 23.03 | 4.617 | 78 | 20.49 | 6.301 | 19.993 | **<0.0001** | **0.023** |
| LOGICAL MEMORY immediate | 740 | 5.13 | 4.008 | 72 | 4.18 | 3.795 | 3.714 | 0.054 | 0.005 |
| LOGICAL MEMORY delayed | 737 | 3.02 | 3.771 | 72 | 3.42 | 3.688 | 0.737 | 0.391 | 0.001 |
| DIGIT SPAN FORWARD LENGTH | 755 | 6.12 | 1.212 | 75 | 4.67 | 1.655 | 90.984 | **<0.0001** | **0.099** |
| DIGIT SPAN BACKWARD LENGTH | 751 | 3.85 | 1.249 | 71 | 2.87 | 1.182 | 40.32 | **<0.0001** | **0.047** |
| ANIMALS 60sec | 780 | 12.22 | 5.234 | 75 | 8.8 | 4.926 | 29.512 | **<0.0001** | **0.033** |
| VEGETABLES 60sec | 773 | 7.62 | 3.786 | 72 | 5.38 | 3.799 | 23.084 | **<0.0001** | **0.027** |
| TRAIL A Seconds | 742 | 68.19 | 39.842 | 72 | 81.24 | 45.818 | 6.847 | 0.009 | 0.008 |
| TRAIL A CORRECT LINES | 330 | 22.87 | 3.571 | 41 | 22.32 | 3.996 | 0.85 | 0.357 | 0.002 |
| TRAIL B Seconds | 612 | 201.13 | 90.181 | 54 | 225.3 | 91.01 | 3.557 | 0.06 | 0.005 |
| TRAIL B  CORRECT LINES | 266 | 19.45 | 7.432 | 29 | 19 | 6.536 | 0.098 | 0.754 | 0 |
| WAIS-R digit symbol | 665 | 26.35 | 13.373 | 66 | 22.42 | 14.778 | 5.079 | 0.025 | 0.007 |
| BOSTON Naming test | 744 | 22.55 | 6.138 | 74 | 17.41 | 8.219 | 44.21 | **<0.0001** | **0.051** |
|  | **Eta squared > 0.1 and p value<0.05 are in bold** | | | | | | |  |  |

|  | Non-visuospatial initial symptom | | | Visuospatial initial symptom | | |  |  |  |
| --- | --- | --- | --- | --- | --- | --- | --- | --- | --- |
|  | **N** | **Mean** | **Std. Deviation** | **N** | **Mean** | **Std. Deviation** | **F** | **Sig.** | **Eta Squared** |
| Age at visit | 862 | 73.9919 | 10.06638 | 28 | 68.0357 | 8.22589 | 9.591 | **0.002** | **0.011** |
| Sex F% | 37.6% | | | 35.7% | | | 0.41 | 1 |  |
| EDUCATION | 856 | 15.49 | 3.105 | 28 | 15.75 | 2.303 | 0.195 | 0.659 | 0 |
| APOEε4% | 61.8% | | | 57.1% | | | 0.25 | 0.62 |  |
| Hachinski score | 840 | 0.81 | 1.24 | 27 | 1.33 | 1.59 | 4.54 | 0.32 | 0.005 |
| MMSE | 827 | 22.85 | 4.812 | 26 | 21.38 | 5.783 | 2.292 | 0.13 | 0.003 |
| LOGICAL MEMORY immediate | 789 | 4.98 | 3.96 | 23 | 7.3 | 4.636 | 7.623 | 0.006 | 0.009 |
| LOGICAL MEMORY delayed | 786 | 3.01 | 3.75 | 23 | 4.7 | 3.913 | 4.53 | 0.034 | 0.006 |
| DIGIT SPAN FORWARD LENGTH | 806 | 6 | 1.326 | 24 | 5.75 | 1.26 | 0.798 | 0.372 | 0.001 |
| DIGIT SPAN BACKWARD LENGTH | 799 | 3.79 | 1.27 | 23 | 3.13 | 1.217 | 5.989 | 0.015 | 0.007 |
| ANIMALS 60sec | 830 | 11.88 | 5.266 | 25 | 13.32 | 6.115 | 1.801 | 0.18 | 0.002 |
| VEGETABLES 60sec | 821 | 7.4 | 3.829 | 24 | 8.17 | 4.104 | 0.92 | 0.338 | 0.001 |
| TRAIL A Seconds | 794 | 68.28 | 39.992 | 20 | 111.6 | 40.753 | 22.873 | **<0.0001** | **0.027** |
| TRAIL A CORRECT LINES | 357 | 22.98 | 3.233 | 14 | 18.43 | 8.064 | 22.556 | **<0.0001** | **0.058** |
| TRAIL B Seconds | 652 | 201.75 | 90.476 | 14 | 265.79 | 63.102 | 6.936 | 0.009 | 0.01 |
| TRAIL B  CORRECT LINES | 286 | 19.42 | 7.311 | 9 | 19.11 | 8.681 | 0.015 | 0.903 | 0 |
| WAIS-R digit symbol | 717 | 26.17 | 13.494 | 14 | 17.29 | 13.624 | 5.947 | 0.015 | 0.008 |
| BOSTON Naming test | 796 | 22.08 | 6.511 | 22 | 22.55 | 6.919 | 0.111 | 0.739 | 0 |
| Eta squared > 0.1 and p value<0.05 are in bold | | | | | | | | | |
