# Supplementary material for: Impact of APOE ε4 genotype on initial cognitive symptoms differs for Alzheimer’s and Lewy body neuropathology
Source: Alzheimers Res Ther. 2021 Jan 23;13:31. doi: 10.1186/s13195-021-00771-1 (PMC7825215; doi:10.1186/s13195-021-00771-1)
Supplement: Supplementary file 4 — Additional file 4: Supplementary Table 4. Coefficients of all logistic regression models. [file 13195_2021_771_MOESM4_ESM.docx]

| **Supplementary Table 1 : Coefficients of all logistic regression models** | | | | |
| --- | --- | --- | --- | --- |
|  | | | | |
| 1. **ADP and LRP alone groups** | |  |  |  |
|  |  |  |  |  |
| 1. **Logistic regression with AD as reference** | | | | |
|  |  |  |  |  |
| **a.i.Amnestic** | |  |  |  |
| Estimate Std. Error z value Pr(>\|z\|) | | | | |
| (Intercept) -4.509005 0.731569 -6.163 7.12e-10 *** | | | | |
| APOE 0.455799 0.154064 2.959 0.00309 ** | | | | |
| Age_visit 0.091613 0.007339 12.483 < 2e-16 *** | | | | |
| SEX -0.285355 0.151149 -1.888 0.05904 . | | | | |
| EDUC -0.041318 0.025668 -1.610 0.10746 | | | | |
| AD_LEWY 3 -0.651022 0.284278 -2.290 0.02202 * | | | | |
| APOE: AD_LEWY 3 -1.060451 0.587923 -1.804 0.07127 . | | | | |
|  |  |  |  |  |
|  |  |  |  |  |
|  |  |  |  |  |
| **a.ii. Executive** | |  |  |  |
| Estimate Std. Error z value Pr(>\|z\|) | | | | |
| (Intercept) 2.459333 1.063908 2.312 0.0208 * | | | | |
| APOE -0.010296 0.243758 -0.042 0.9663 | | | | |
| Age_visit -0.065130 0.010215 -6.376 1.82e-10 *** | | | | |
| SEX -0.314754 0.243066 -1.295 0.1953 | | | | |
| EDUC -0.002992 0.039756 -0.075 0.9400 | | | | |
| AD_LEWY 3 0.682509 0.406135 1.680 0.0929 . | | | | |
| APOE:AD_LEWY 3 -0.885315 1.127899 -0.785 0.4325 | | | | |
|  |  |  |  |  |
|  |  |  |  |  |
|  |  |  |  |  |
| **a.iii. Language** |  |  |  |  |
| Estimate Std. Error z value Pr(>\|z\|) | | | | |
| (Intercept) 1.38395 0.83617 1.655 0.09790 . | | | | |
| APOE -0.39893 0.18359 -2.173 0.02978 * | | | | |
| Age_visit -0.06947 0.00799 -8.695 < 2e-16 *** | | | | |
| SEX 0.53865 0.17937 3.003 0.00267 ** | | | | |
| EDUC 0.06872 0.03119 2.203 0.02756 * | | | | |
| AD_LEWY 3 0.63887 0.31738 2.013 0.04412 * | | | | |
| APOE:AD_LEWY 3 -1.21196 1.09382 -1.108 0.26786 | | | | |
|  |  |  |  |  |
|  |  |  |  |  |
| **a.iv. Visual spatial** | |  |  |  |
| Estimate Std. Error z value Pr(>\|z\|) | | | | |
| (Intercept) 2.43801 1.56384 1.559 0.1190 | | | | |
| APOE -0.78165 0.40195 -1.945 0.0518 . | | | | |
| Age_visit -0.08254 0.01489 -5.542 2.99e-08 *** | | | | |
| SEX -0.01244 0.37069 -0.034 0.9732 | | | | |
| EDUC 0.00325 0.06234 0.052 0.9584 | | | | |
| AD_LEWY 3 -0.24745 0.76330 -0.324 0.7458 | | | | |
| APOE:AD_LEWY 3 4.15093 0.97516 4.257 2.08e-05 *** | | | | |
|  |  |  |  |  |
|  |  |  |  |  |
|  | |  |  |  |
| 1. **Logistic regression with DLB as reference** | | | | |
|  |  |  |  |  |
| **b.i.Amnestic** | |  |  |  |
| Estimate Std. Error z value Pr(>\|z\|) | | | | |
| (Intercept) -5.811049 0.875922 -6.634 3.26e-11 *** | | | | |
| APOE -1.665102 1.144416 -1.455 0.1457 | | | | |
| Age_visit 0.091613 0.007339 12.483 < 2e-16 *** | | | | |
| SEX -0.285355 0.151149 -1.888 0.0590 . | | | | |
| EDUC -0.041318 0.025668 -1.610 0.1075 | | | | |
| AD_LEWY 0.651022 0.284278 2.290 0.0220 * | | | | |
| APOE:AD_LEWY 1.060451 0.587923 1.804 0.0713 . | | | | |
|  |  |  |  |  |
| **b.ii.Executive** | |  |  |  |
| Estimate Std. Error z value Pr(>\|z\|) | | | | |
| (Intercept) 3.824351 1.271209 3.008 0.00263 ** | | | | |
| APOE -1.780925 2.215885 -0.804 0.42157 | | | | |
| Age_visit -0.065130 0.010215 -6.376 1.82e-10 *** | | | | |
| SEX -0.314754 0.243066 -1.295 0.19534 | | | | |
| EDUC -0.002992 0.039756 -0.075 0.94002 | | | | |
| AD_LEWY -0.682509 0.406135 -1.680 0.09286 . | | | | |
| APOE:AD_LEWY 0.885315 1.127899 0.785 0.43250 | | | | |
|  |  |  |  |  |
| **b.iii.Language** |  |  |  |  |
| Estimate Std. Error z value Pr(>\|z\|) | | | | |
| (Intercept) 2.66168 0.98710 2.696 0.00701 ** | | | | |
| APOE -2.82286 2.16421 -1.304 0.19212 | | | | |
| Age_visit -0.06947 0.00799 -8.695 < 2e-16 *** | | | | |
| SEX 0.53865 0.17937 3.003 0.00267 ** | | | | |
| EDUC 0.06872 0.03119 2.203 0.02756 * | | | | |
| AD_LEWY -0.63887 0.31738 -2.013 0.04412 * | | | | |
| APOE:AD_LEWY 1.21196 1.09382 1.108 0.26786 | | | | |
|  |  |  |  |  |
|  |  |  |  |  |
| **b.iv.Visualspatial** | |  |  |  |
| Estimate Std. Error z value Pr(>\|z\|) | | | | |
| (Intercept) 1.94311 2.11481 0.919 0.358 | | | | |
| APOE 7.52022 1.82395 4.123 3.74e-05 *** | | | | |
| Age_visit -0.08254 0.01489 -5.542 2.99e-08 *** | | | | |
| SEX -0.01244 0.37069 -0.034 0.973 | | | | |
| EDUC 0.00325 0.06234 0.052 0.958 | | | | |
| AD_LEWY 0.24745 0.76330 0.324 0.746 | | | | |
| APOE:AD_LEWY -4.15093 0.97516 -4.257 2.08e-05 *** | | | | |
| Significance. codes: 0 ‘***’ 0.001 ‘**’ 0.01 ‘*’ 0.05 ‘.’ 0.1 ‘ ’ 1 | | | | |
|  |  |  |  |  |

| 1. **ADP and ADP-LRP groups (ADP-LRP coded as DLB-AD for ease of reading analysis summary)** | | |  |  |
| --- | --- | --- | --- | --- |
|  |  |  |  |  |
| 1. **Logistic regression with ADP as reference** | | | | |
|  |  |  |  |  |
| **c.i.Amnestic** |  |  |  |  |
| Estimate Std. Error z value Pr(>\|z\|) | | | | |
| (Intercept) -3.117091 0.566725 -5.500 3.79e-08 *** | | | | |
| APOE 0.619522 0.183827 3.370 0.000751 *** | | | | |
| Age_visit 0.071742 0.005573 12.873 < 2e-16 *** | | | | |
| SEX 0.070611 0.120803 0.585 0.558877 | | | | |
| EDUC -0.065875 0.020179 -3.265 0.001096 ** | | | | |
| AD_DLB-AD 2.00 -0.064626 0.174188 -0.371 0.710629 | | | | |
| APOE:AD_DLB-AD 2.00 -0.207761 0.236364 -0.879 0.379407 | | | | |
| --- |  |  |  |  |
|  |  |  |  |  |
|  |  |  |  |  |
| **c.ii.Executive** | |  |  |  |
| Estimate Std. Error z value Pr(>\|z\|) | | | | |
| (Intercept) 1.687228 0.845080 1.997 0.0459 * | | | | |
| APOE -0.132171 0.282136 -0.468 0.6395 | | | | |
| Age_visit -0.054053 0.008151 -6.632 3.32e-11 *** | | | | |
| SEX -0.428309 0.197202 -2.172 0.0299 * | | | | |
| EDUC 0.010639 0.031122 0.342 0.7325 | | | | |
| AD_DLB-AD 2.00 -0.058729 0.282881 -0.208 0.8355 | | | | |
| APOE:AD_DLB-AD 2.00 0.115855 0.371715 0.312 0.7553 | | | | |
|  |  |  |  |  |
|  |  |  |  |  |
|  |  |  |  |  |
| **c.iii.Language** |  |  |  |  |
| Estimate Std. Error z value Pr(>\|z\|) | | | | |
| (Intercept) 0.412103 0.686657 0.600 0.548401 | | | | |
| APOE -0.819707 0.241323 -3.397 0.000682 *** | | | | |
| Age_visit -0.055183 0.006475 -8.522 < 2e-16 *** | | | | |
| SEX 0.134151 0.149866 0.895 0.370715 | | | | |
| EDUC 0.095034 0.025746 3.691 0.000223 *** | | | | |
| AD_DLB-AD 2.00 0.119500 0.210351 0.568 0.569970 | | | | |
| APOE:AD_DLB-AD 2.00 0.445061 0.301276 1.477 0.139607 | | | | |
|  |  |  |  |  |
|  |  |  |  |  |
| **c.iv.Visual Spatial** | |  |  |  |
| Estimate Std. Error z value Pr(>\|z\|) | | | | |
| (Intercept) 1.35756 1.21218 1.120 0.263 | | | | |
| APOE -0.24519 0.39491 -0.621 0.535 | | | | |
| Age_visit -0.07177 0.01155 -6.213 5.21e-10 *** | | | | |
| SEX -0.05829 0.28495 -0.205 0.838 | | | | |
| EDUC 0.03131 0.04784 0.654 0.513 | | | | |
| AD_DLB-AD 2.00 -0.00789 0.38371 -0.021 0.984 | | | | |
| APOE:AD_DLB-AD 2.00 -0.55237 0.56123 -0.984 0.325 | | | | |
|  |  |  |  |  |
|  | | |  |  |
| 1. **Logistic regression with ADP-LRP(DLB-AD mixed) as reference** | | | | |
|  |  |  |  |  |
| **d.i.Amnestic** | |  |  |  |
| Estimate Std. Error z value Pr(>\|z\|) | | | | |
| (Intercept) -3.052465 0.615380 -4.960 7.04e-07 *** | | | | |
| APOE 0.827284 0.396233 2.088 0.0368 * | | | | |
| Age_visit 0.071742 0.005573 12.873 < 2e-16 *** | | | | |
| SEX 0.070611 0.120803 0.585 0.5589 | | | | |
| EDUC -0.065875 0.020179 -3.265 0.0011 ** | | | | |
| AD_DLB-AD -0.064626 0.174188 -0.371 0.7106 | | | | |
| APOE:AD_DLB-AD -0.207761 0.236364 -0.879 0.3794 | | | | |
|  |  |  |  |  |
|  |  |  |  |  |
| **d.ii.Executive** | |  |  |  |
| Estimate Std. Error z value Pr(>\|z\|) | | | | |
| (Intercept) 1.745957 0.930450 1.876 0.0606 . | | | | |
| APOE -0.248026 0.613985 -0.404 0.6862 | | | | |
| Age_visit -0.054053 0.008151 -6.632 3.32e-11 *** | | | | |
| SEX -0.428309 0.197202 -2.172 0.0299 * | | | | |
| EDUC 0.010639 0.031122 0.342 0.7325 | | | | |
| AD_DLB-AD -0.058729 0.282881 -0.208 0.8355 | | | | |
| APOE:AD_DLB-AD 0.115855 0.371715 0.312 0.7553 | | | | |
|  |  |  |  |  |
| **d.iii.Language** |  |  |  |  |
| Estimate Std. Error z value Pr(>\|z\|) | | | | |
| (Intercept) 0.292604 0.745460 0.393 0.694678 | | | | |
| APOE -1.264769 0.515144 -2.455 0.014082 * | | | | |
| Age_visit -0.055183 0.006475 -8.522 < 2e-16 *** | | | | |
| SEX 0.134151 0.149866 0.895 0.370715 | | | | |
| EDUC 0.095034 0.025746 3.691 0.000223 *** | | | | |
| AD_DLB-AD 0.119500 0.210351 0.568 0.569970 | | | | |
| APOE:AD_DLB-AD 0.445061 0.301276 1.477 0.139607 | | | | |
|  |  |  |  |  |
|  |  |  |  |  |
| **e.iv.Visualspatial** | |  |  |  |
| Estimate Std. Error z value Pr(>\|z\|) | | | | |
| (Intercept) 1.36545 1.32028 1.034 0.301 | | | | |
| APOE 0.30718 0.88450 0.347 0.728 | | | | |
| Age_visit -0.07177 0.01155 -6.213 5.21e-10 *** | | | | |
| SEX -0.05829 0.28495 -0.205 0.838 | | | | |
| EDUC 0.03131 0.04784 0.654 0.513 | | | | |
| AD_DLB-AD -0.00789 0.38371 -0.021 0.984 | | | | |
| APOE:AD_DLB-AD -0.55237 0.56123 -0.984 0.325 | | | | |
| Significance. codes: 0 ‘***’ 0.001 ‘**’ 0.01 ‘*’ 0.05 ‘.’ 0.1 ‘ ’ 1 | | | | |
|  |  |  |  |  |
|  |  |  |  |  |
|  |  |  |  |  |
