# Supplementary material for: Impact of APOE ε4 genotype on initial cognitive symptoms differs for Alzheimer’s and Lewy body neuropathology
Source: Alzheimers Res Ther. 2021 Jan 23;13:31. doi: 10.1186/s13195-021-00771-1 (PMC7825215; doi:10.1186/s13195-021-00771-1)
Supplement: Supplementary file 5 — Additional file 5: Supplementary material. [file 13195_2021_771_MOESM5_ESM.docx]

**Supplementary material**

1. Vascular co-pathology in the three groups ADP, LRP and ADP-LRP

The NACC Neuropathology Data Set has undergone several revisions since its inception in 2002, the most substantial of which occurred in 2014. Over this time the data regarding characterization of the degree of vascular pathology has changed. In order to standardize the characterization of vascular pathology, in the current version, presence of one or more ischemic, hemorrhagic, or vascular pathology (including mild severity indicated for pathologies such as atherosclerosis) is documented as present, absent or unknown.

This makes it difficult to accurately find correlation values between ante-mortem Hachinski score at initial visit and categorical variable of presence versus absence of vascular pathology at autopsy.

| **Pathology** | **Hachinski score**  **Mean (SD)** | **N** | **% Vascular pathology** | **N** |
| --- | --- | --- | --- | --- |
| **ADP** | 0.98 (1.40) | 1278 | 98.40% | 1292 |
| **LRP** | 1.14(1.80) | 88 | 91.10% | 85 |
| **ADP-LRP** | 0.84(1.30) | 871 | 98.20% | 892 |

2. The baseline cognitive status of subjects at initial visit within each neuropathology group evaluated was captured by CDR-Global scores (0=unimpaired, 0.5=mild cognitive impairment and 1= early dementia). The number of subjects by CDR-G status is noted below.

| **Pathology** | **CDR-G=0** | **CDR-G=0.5** | **CDR-G=1** | **Total N** |
| --- | --- | --- | --- | --- |
| **ADP** | 24 | 688 | 591 | 1303 |
| **LRP** | 4 | 56 | 30 | 90 |
| **ADP-LRP** | 7 | 428 | 460 | 895 |

3. The number of subjects across the three neuropathology groups by the number of *APOE*-ε4 alleles

|  | **Number of *APOE*-ε4** **alleles** | | |  |
| --- | --- | --- | --- | --- |
| **Pathology** | **0** | **1** | **2** | **Total** |
| **ADP** | 617 | 546 | 140 | 1303 |
| **LRP** | 72 | 16 | 2 | 90 |
| **ADP-LRP** | 344 | 409 | 142 | 895 |

4. Comparative analysis of two logistic regression models using the 3-level *APOE*-ε4 dose (0, 1,&2) versus 2-level *APOE*-ε4 presence/absence for ADP versus ADP-LRP

Vuong test comparing both logistic models evaluating ADP versus ADP-LRP(mixed)

Model 1= *APOE*-ε4 is denoted as levels 0,1 and 2 (*APOE*-ε4 dose)

Model 2= *APOE*-ε4 is denoted as levels 0 and 1 (presence/absence)

Vuong Non-Nested Hypothesis Test-Statistic:

(test-statistic is asymptotically distributed N(0,1) under the null that the models are indistinguishable)

Amnestic:

-------------------------------------------------------------

Vuong z-statistic H_A p-value

Raw 0.8937062 model1 > model2 0.18574

AIC-corrected 0.8937062 model1 > model2 0.18574

BIC-corrected 0.8937062 model1 > model2 0.18574

Executive:

-------------------------------------------------------------

Vuong z-statistic H_A p-value

Raw 0.7957577 model1 > model2 0.21309

AIC-corrected 0.7957577 model1 > model2 0.21309

BIC-corrected 0.7957577 model1 > model2 0.21309

Language

-------------------------------------------------------------

Vuong z-statistic H_A p-value

Raw 0.8712564 model1 > model2 0.19181

AIC-corrected 0.8712564 model1 > model2 0.19181

BIC-corrected 0.8712564 model1 > model2 0.19181

Visuospatial:

-------------------------------------------------------------

Vuong z-statistic H_A p-value

Raw -1.086358 model2 > model1 0.13866

AIC-corrected -1.086358 model2 > model1 0.13866

BIC-corrected -1.086358 model2 > model1 0.13866
